# Pregnancy and perinatal outcomes in pregnancies resulting from time interval between a freeze-all cycle and a subsequent frozen-thawed single blastocyst transfer

**DOI:** 10.1186/s12884-020-02858-3

**Published:** 2020-03-14

**Authors:** Shiqiao Hu, Bei Xu, Rui Long, Lei Jin

**Affiliations:** Department of Reproductive Medicine Center, Tongji Hospital, Tongji Medical College, Huazhong University of Science and Technology, 1095 JieFang Avenue, Wuhan, 430030 People’s Republic of China

**Keywords:** Freeze-all strategy, Perinatal outcomes, Time interval, Macrosomia

## Abstract

**Background:**

Adverse obstetric outcomes are correlated with altered circulating hormone levels at the time implantation by the trophectoderm. What’ more, embryo freezing process may also have adverse effect on perinatal outcomes. This study aims to evaluate whether increasing interval time between a freeze-all cycle and a subsequent frozen-thawed single blastocyst transfer could have any effect on pregnancy and perinatal outcomes.

**Methods:**

This was a retrospective cohort study included the first single blastocyst transfer in artificially cycles of all patients who underwent a freeze-all cycle between January 1^st^, 2016 and September 30^th^, 2018. All patients were divided into two groups according to the time interval between oocyte retrieval and the day of first frozen-thawed embryo transferred (FET): Group 1 (immediate FET cycles) and Group 2 (delayed FET cycles).

**Results:**

No significant differences were reported between the two groups regarding the rates of clinical pregnancy, live birth, biochemical pregnancy and pregnancy loss even after adjusting for measured confounding. When accounting for perinatal outcomes, gestational age, birth weight, delivery mode, fetus gender, preterm birth, gestational hypertension, GDM, placenta previa, fetal malformation and low birthweight also did not vary significantly between the two groups. Only the incidence of macrosomia was more frequently in the Group 2 compared with the Group 1 (AOR 3.886, 95%CI 1.153–13.103, *P* = 0.029) after adjusting with a multiple logistic regression model.

**Conclusions:**

We found delayed FET cycles for blastocyst transfer following freeze-all cycles may not improve the pregnancy outcomes. On the contrary, postponement of FET cycles may increase the risk of macrosomia. Therefore, FET cycles for blastocyst transfer should be done immediately to avoid adverse effects of delayed time on perinatal outcomes.

## Background

Since the first baby with frozen-thawed embryo transferred (FET) was born in 1983, the embryo cryopreservation technology has progressively increased [1]. As is well-known, its widespread application is due to multi-follicular stimulation which may enable excessive number of oocytes to be obtained and eventually increase the cumulative live birth rate [2, 3]. With the increased safety and efficacy of embryo cryopreservation technology, the concept of freeze-all strategy has evolved due to reduce the risk of ovarian hyperstimulation syndrome (OHSS) and improve endometrial environment [4–7]. During the process of in-vitro fertilization (IVF), controlled ovarian hyperstimulation (COH) is a double-edged sword. COH can increase the number of oocytes to be retrieved and enhance cumulative pregnancy rate, but at the same time leads to supraphysiological hormone levels. Embryo implantation and subsequent placental growth and maintenance is correlated with altered circulating hormone levels at the time implantation by the trophectoderm [8]. It affected not only the endometrial receptivity and early implantation but also on placentation and subsequent fetal growth [9–12]. Some studies clearly demonstrated that the increased risk of developing disorders related to abnormal placentation in patients with elevated estradiol (E_2_) levels and suggested that ovarian hyperstimulation may change angiogenesis of the endometrium [8, 10, 13, 14]. Although the detrimental effect of supraphysiological hormone levels on pregnancy and perinatal outcomes seems clear, our concerns are how long it takes for the endometrium after COH return to pre-stimulation functionality and whether supraphysiological hormone levels could have negative effect on a subsequent treatment so that FET need to be postponed in an attempt. Meanwhile, embryo freezing process may also have adverse effect on perinatal outcomes [15, 16]. Therefore, we performed this study to evaluate whether increasing interval time between a freeze-all cycle and a subsequent FET could have any effect on pregnancy and perinatal outcomes.

## Methods

### Study population and design

We conducted a retrospective cohort study including all patients (≤40 years old) who underwent a freeze-all IVF or intracytoplasmic sperm injection (ICSI) cycle and subsequent first single blastocyst transfer in artificially cycles at the Reproductive Medical Center of Tongji hospital between January 1^st^, 2016 and September 30^th^, 2018. Only the outcomes of the first FET cycles performed after a freeze-all IVF or ICSI cycle with GnRH agonists or antagonists were assessed. The indications for a freeze-all cycle are as following: high progesterone concentration (> 1.5 ng/ml), prevention of OHSS (the number of oocytes retrieved > 20 or high estradiol concentration > 7000 pg/ml), hydrosalpinx (diameter > 3 cm), inappropriate endometrium environment. Exclusion criteria were: 1) blastocyst biopsy for preimplantation genetic diagnosis (PGD) or preimplantation genetic screening (PGS); 2) multiple COH cycle before FET; 3) frozen or donated oocytes; 4) patients with hypertension, diabetes mellitus, abnormal glucose tolerance or insulin resistance; 5) use GnRH agonist during frozen-thawed cycle. 6) uterine malformation.

### Protocol of COH during freeze-all cycles

Conventional IVF or ICSI was conducted for all patients. The protocol of COH was determined individually combination with GnRH agonists or antagonists. Serial vaginal ultrasonography was used to observe ovarian response. When two leading follicles reached mean diameter ≥ 18 mm, HCG (10,000 IU, EMD Serono) was then used to trigger ovulation. On the day of HCG injection, serum concentrations of E_2_ and progesterone were measured using an Immulite Automated Analyzer System (ECL2012, Siemens, Germany). Oocytes were obtained transvaginally 34–36 h after HCG injection [17].

### Embryo culture, vitrification and warming

In IVF cycles, every oocyte was inseminated with 10,000 motile spermatozoa after 4 h oocytes retrieval. However, patients with severe oligospermia or difficult fertilization received ICSI. Then, fertilized oocytes were continuously cultured in G1 medium for 2 more days. All of the embryos from IVF or ICSI were checked on the morning of day 3 after oocyte retrieval (approximately 69 h after initial insemination) [18]. No embryo was transferred in fresh cycle. All available embryos were cryopreserved for subsequent frozen-thawed cycles and cryopreserved by vitrification [19]. Embryos vitrification were using the Cryotop device and commercially available vitrification solutions (Kitazato, Japan) and full-to-expanded blastocysts on day 5 or 7 during embryo culture. The best one blastocyst was warmed on the day of embryo transfer. During the warming procedure, vitrified embryos were warmed to 37 °C using a vitrification–warming kit. Warmed blastocyst was then cultured for at least 2 h prior to further evaluation [18].

### Endometrial preparation and embryos transfer

FET cycles for endometrium preparation was an artificially supplemented cycle monitored by vaginal ultrasonography. Estradiol valerate (Progynova, Germany) was administered orally at a dose of 2 mg twice daily from day 2 to day 10 of the menstrual cycle until the endometrium thickness exceeded 7 mm, and then 40 mg of progesterone intramuscularly and 20 mg of progesterone orally was given daily. Embryos transfer was performed according to the protocol described above except for the day of transfer. Only one blastocyst was transferred.

### Definition of time interval

**Fig. 1:**
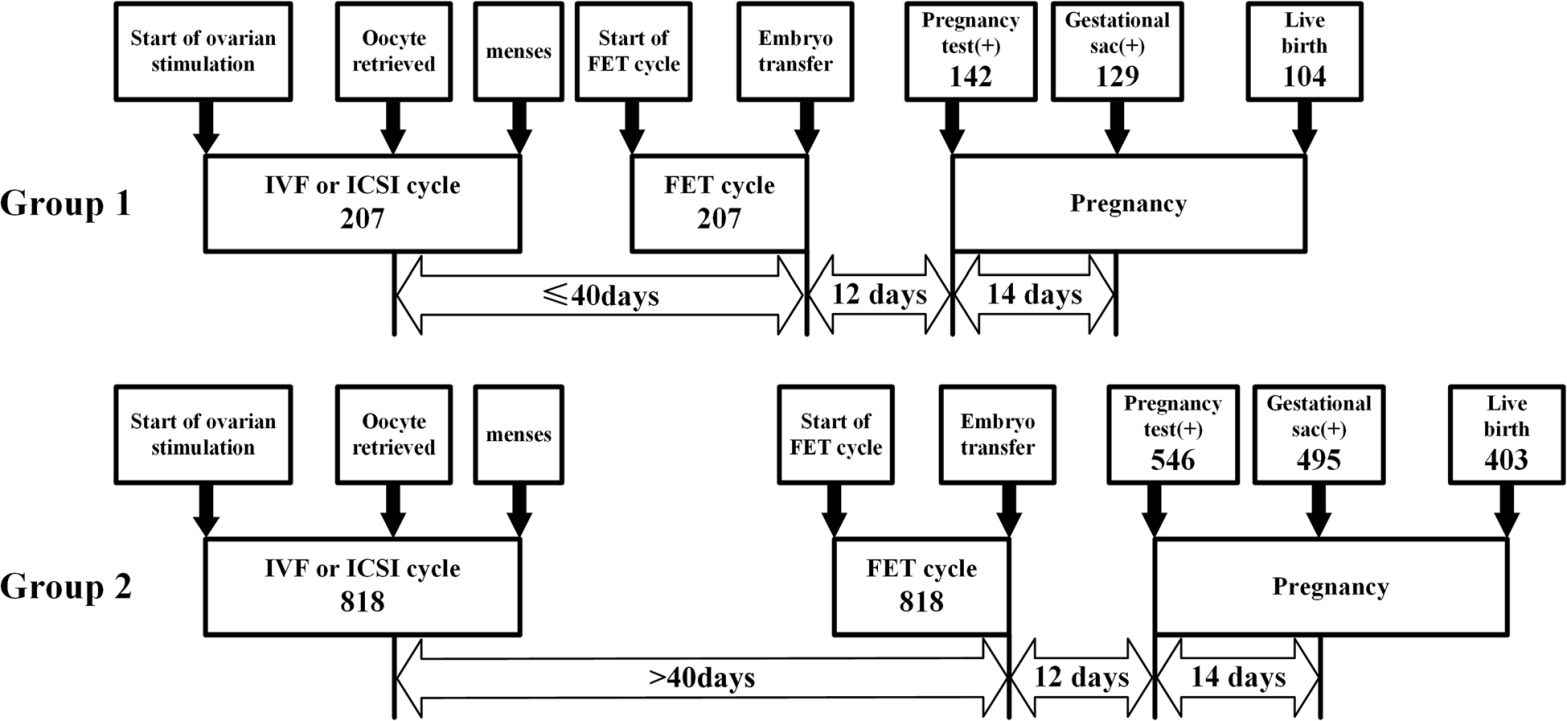
The design of study. Note: The FET cycles were divided into two group: Group 1 (immediate FET cycles) and Group 2 (delayed FET cycles)

The time interval between a freeze-all cycle and the first FET cycle depended on the time interval between oocyte retrieval and the day of first frozen-thawed blastocyst transferred. As shown in Fig. 1, we divided all patients into two groups: Group 1, ≤40 days after oocyte retrieval; Group 2, more than 40 days after oocyte retrieval. This cutoff was devised by adding the interval between menstrual cycle (28–35 days) and an extra interval for embryo culture (5–6 days) and finally we chose 40 days as a cutoff value. If the patients were assigned to the Group 1, they had an immediate FET. Otherwise, they had a delayed FET in the Group 2.

### Main outcome measure and statistical analysis

Basic demographic characteristics were compared between the two groups with the use of Mann-Whitney (for continuous variables) and chi square tests or Fisher exact tests (for categoric variables). Except for basic demographic characteristics, the rates of clinical pregnancy, biochemical pregnancy, live birth and pregnancy loss were also included in the pregnancy outcomes. A multiple logistic regression analysis was performed to compare the association between the two groups.

For the patients with singleton live birth, perinatal outcomes were the main outcomes of our study which included gestational age, birth weight, fetus gender, preterm birth (< 37 weeks), gestational hypertension, gestational diabetes mellitus (GDM), placenta previa, fetal malformation, macrosomia (≥4000 g) and low birthweight (< 2500 g). A multiple logistic regression analysis was also used to compare correlations.

## Results

A total of 1025 FET cycles performed following a freeze-all IVF or ICSI cycle were included in the analysis. All patients were divided into two groups: Group 1 with 207 FET cycles; Group 2 with 818 FET cycles. The majority of FET cycles were initiated after more than a menstrual cycle (79.8%).

### Patients general characteristics in freeze-all cycles

Patients general characteristics in freeze-all cycles between two groups were showed in Table 1. No significant differences were found between the two groups with regard to BMI, baseline FSH, AFC, infertility years, gonadotropins dose and number of oocytes retrieved. What’ more, the rates of patients with > 20 oocytes retrieved and progesterone > 1.5 ng/ml were also comparable in the two groups. However, age, infertility diagnosis, method of fertilization, duration of stimulation, protocol for COH, E_2_ and progesterone levels, and endometrial thickness were all significantly different between the two groups. When accounting for the indication for IVF/ICSI, there were no significant differences between the two groups with regard to the rates of pelvic and tubal factor, endometriosis and PCOS. The rate of male infertility factor was significantly higher in the Group 2.

**Table 1:** Patients general characteristics between the two groups in freeze-all cycles

	Group 1 (207)	Group 2 (818)	P
Age (year)	29.75 ± 4.08	30.57 ± 4.04	0.007*
BMI (kg/m^2^)	21.60 ± 2.91	21.57 ± 2.86	0.897
Baseline FSH (mIU/ml)	7.04 ± 1.72	7.23 ± 2.05	0.185
AFC	15.57 ± 7.01	15.50 ± 6.87	0.952
Duration of infertility years (year)	3.50 ± 2.35	3.24 ± 2.34	0.073
Mean interval day	28.75 ± 3.47	93.98 ± 58.98	< 0.001*
Infertility diagnosis			0.013*
Primary infertility	141 (68.1%)	480 (58.7%)	
Secondary infertility	66 (31.9%)	338 (41.3%)	
The indications for IVF/ICSI			
Pelvic and tubal factor	113 (54.6%)	461 (56.4%)	0.647
Male infertility	61 (29.5%)	313 (38.3%)	0.019*
Endometriosis	10 (4.8%)	65 (7.9%)	0.124
PCOS	25 (12.1%)	112 (13.7%)	0.542
Method of fertilization			0.030*
IVF	125 (60.4%)	559 (68.3%)	
ICSI	82 (39.6%)	259 (31.7%)	
Duration of stimulation (day)	11.0 ± 1.71	10.55 ± 1.90	< 0.001*
Gonadotropins dose (IU)	2308.15 ± 911.18	2261.71 ± 820.78	0.901
E_2_ on HCG (pg/ml)	3867.84 ± 2140.15	4397.73 ± 2324.80	0.001*
Number of oocytes retrieved	18.23 ± 7.65	17.74 ± 7.65	0.445
Progesterone on HCG (ng/ml)	1.24 ± 0.69	1.34 ± 0.65	0.004*
Endometrial thickness (mm)	12.38 ± 2.87	11.60 ± 2.62	< 0.001*
Protocol for COH			0.013*
Agonist	175 (84.5%)	626 (76.5%)	
Antagonist	32 (15.5%)	192 (23.5%)	
> 20 Oocytes retrieved	82 (39.6%)	331 (40.5%)	0.824
Progesterone> 1.5 ng/ml	64 (30.9%)	273 (33.4%)	0.502

Note: 1025 freeze-all cycles were included. Continuous data: mean ± SD. Categorical data: n (rate). Mann-Whitney tests were used in the continuous data and chi square tests were used in the categorical data. *, *P*<0.05

*COH* controlled ovarian hyperstimulation, *PCOS* polycystic ovary syndrome

*AFC* antral follicle count, *IVF* in vitro fertilization, *ICSI* intracytoplasmic sperm injection

### Relationship between time interval and FET pregnancy outcomes

Patients baseline characteristics and pregnancy outcomes regarding the FET cycles were reported in Table 2. For the baseline characteristics, only age and method of fertilization were significantly different between the two groups. For the pregnancy outcomes, no significant differences were reported between the two groups regarding the rates of clinical pregnancy, biochemical pregnancy, live birth and pregnancy loss. Therefore, in order to eliminate the influence of baseline characteristics on the pregnancy outcomes, we performed a separate multiple logistic regression model for each pregnancy outcome by adjusting for age, BMI, endometrial thickness, ICSI, male infertility, endometriosis, PCOS, protocol for COH, > 20 oocytes retrieved and progesterone > 1.5 ng/ml (Table 3). No association was found between the two groups in the rates of clinical pregnancy, biochemical pregnancy, live birth and pregnancy loss.

**Table 2:** Patients characteristics and pregnancy outcomes between the two groups in FET cycles

	Group 1 (207)	Group 2 (818)	P
Clinical pregnancy	129 (62.3%)	495 (60.5%)	0.634
Biochemical pregnancy	13 (6.3%)	51 (6.2%)	0.981
Live birth	104 (50.2%)	403 (49.3%)	0.802
Pregnancy loss	25 (12.1%)	92 (11.2%)	0.737
Age	29.82 ± 4.09	30.83 ± 4.04	0.001*
BMI	21.60 ± 2.91	21.56 ± 2.85	0.905
Endometrial thickness (mm)	9.35 ± 1.43	9.32 ± 1.53	0.932
Method of fertilization			0.030*
IVF	125 (60.4%)	559 (68.3%)	
ICSI	82 (39.6%)	259 (31.7%)	

Note: 1025 FET cycles were included. Continuous data: mean ± SD. Categorical data: n (rate). Mann-Whitney tests were used in the continuous data and chi square tests were used in the categorical data. *, *P*<0.05

*IVF* in vitro fertilization, *ICSI* intracytoplasmic sperm injection, *FET* frozen-thawed embryo transfer

**Table 3:** The association between the two groups in pregnancy outcomes

	OR (95%CI)	AOR (95%CI)	P(a)
Clinical pregnancy	0.927 (0.677–1.269)	1.008 (0.730–1.393)	0.960
Biochemical pregnancy	0.992 (0.529–1.861)	1.019 (0.536–1.938)	0.954
Live birth	0.962 (0.709–1.305)	1.051 (0.767–1.440)	0.758
Pregnancy loss	0.923 (0.576–1.477)	0.882 (0.545–1.427)	0.609

Note: Each pregnancy outcome between the two groups was adjusted for age, *BMI* endometrial thickness, *ICSI* male infertility, endometriosis, *PCOS* protocol for COH, > 20 oocytes retrieved and progesterone > 1.5 ng/ml based on a multiple logistic regression model. *OR* odd ratio, *AOR* adjusted odd ratio

### Relationship between time interval and FET perinatal outcomes

**Fig. 2:**
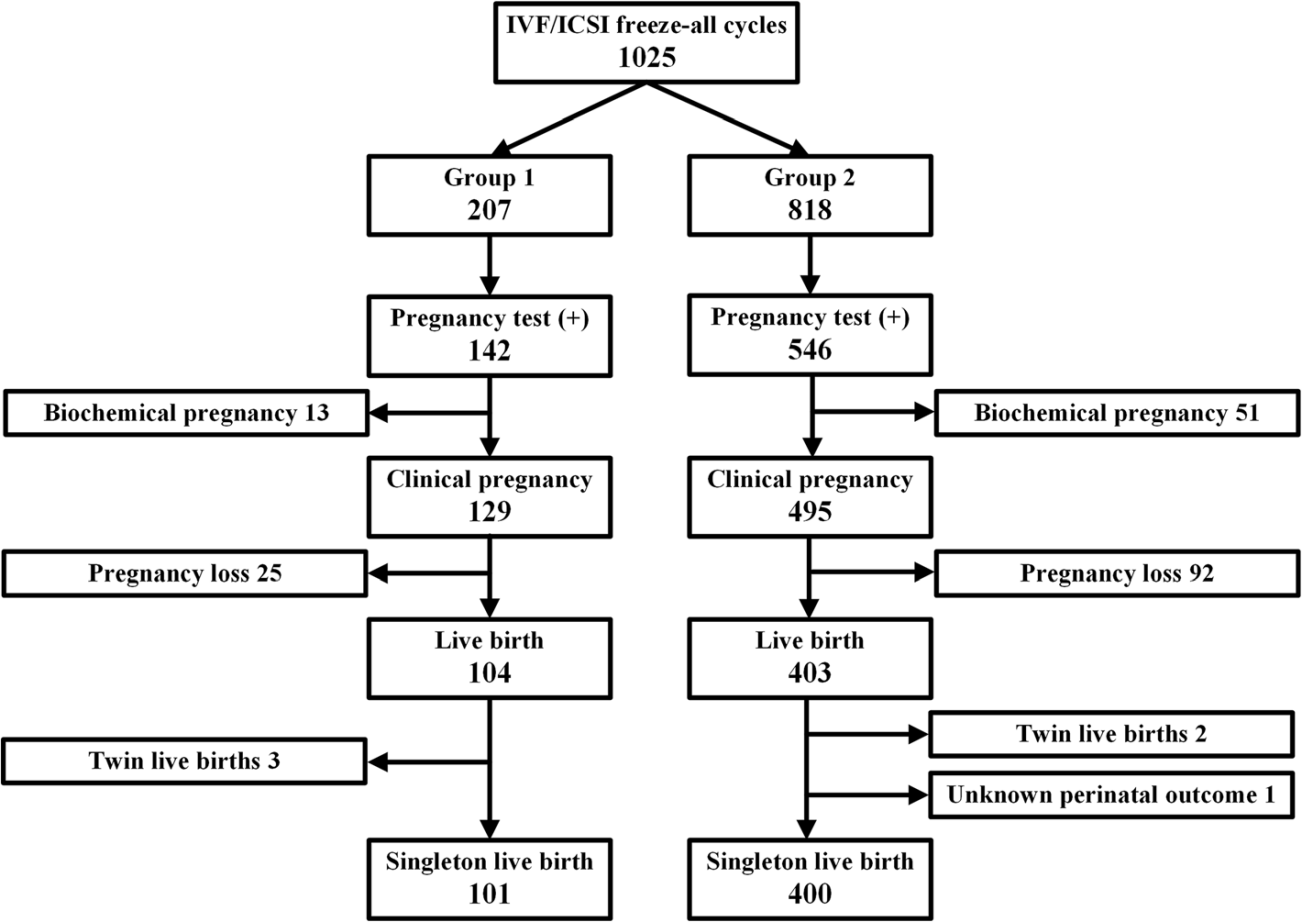
The selection process for singleton live birth

Of all 1025 cycles in our study, there were 507 FET cycles with live birth. As shown in Fig. 2, three patients with twin live births were excluded in the Group 1, and two patients with twin live births and one patient without perinatal outcome were excluded in the Group 2. Finally, 501 FET cycles with singleton live birth were included in the analysis. Further details regarding the patients with singleton live birth were presented in Table 4. Except for the rate of male infertility factor, BMI, endometrial thickness and others indications for IVF/ICSI did not differ significantly between the two groups. However, age and method of fertilization were significantly different. When accounting for perinatal outcomes, no significant differences were found regarding gestational age, birth weight, delivery mode, fetus gender. The incidence of preterm birth (< 37 weeks), gestational hypertension, GDM, placenta previa, fetal malformation and low birthweight (< 2500 g) were also comparable between the two groups. Only the incidence of macrosomia (≥4000 g) was significantly higher in the Group 2 compared with the Group 1 (3.0% in Group 1 versus 10.5% in Group 2). To further clarify the association between time interval and perinatal outcomes, as shown in Table 5, the incidence of caesarean delivery, male fetus, preterm birth, gestational hypertension, GDM, placenta previa, fetal malformation and low birthweight did not vary significantly between the two groups, even after using a multiple logistic regression model. Only the incidence of macrosomia was more frequently in the Group 2 compared with the Group 1 (AOR 3.886, 95%CI 1.153–13.103, *P* = 0.029).

**Table 4:** Patients characteristics and perinatal outcomes between the two groups with singleton live birth

	Group 1 (101)	Group 2 (400)	P
Age (year)	29.30 ± 3.56	30.39 ± 3.90	0.018*
BMI (kg/m^2^)	21.71 ± 3.02	21.60 ± 2.82	0.937
Endometrial thickness (mm)	9.41 ± 1.61	9.44 ± 1.41	0.394
The indications for IVF/ICSI			
Pelvic and tubal factor	53 (52.5%)	227 (56.8%)	0.439
Male infertility	29 (28.7%)	164 (41.0%)	0.023*
Endometriosis	3 (3.0%)	33 (8.3%)	0.066
PCOS	19 (18.8%)	53 (13.3%)	0.155
> 20 oocytes retrieved	44 (43.6%)	176 (44.0%)	0.937
progesterone> 1.5 ng/ml	36 (35.6%)	123 (30.8%)	0.345
Protocol for COH			0.096
Agonist	87 (86.1%)	315 (78.8%)	
Antagonist	14 (13.9%)	85 (21.2%)	
Method of fertilization			0.025*
IVF	54 (53.5%)	262 (65.5%)	
ICSI	47 (46.5%)	138 (34.5%)	
Gestational age (day)	272.13 ± 8.09	271.75 ± 10.88	0.928
Birth weight(g)	3327.43 ± 456.84	3372.40 ± 519.11	0.456
Delivery mode			0.513
Natural labor	10 (9.9%)	49 (12.3%)	
Caesarean delivery	91 (90.1%)	351 (87.7%)	
Gender			0.507
Male	63 (62.4%)	235 (58.8%)	
Female	38 (37.6%)	165 (41.2%)	
Abnormal perinatal outcomes			
Preterm birth<37 weeks	4 (4.0%)	29 (7.3%)	0.234
Gestational hypertension	7 (6.9%)	29 (7.3%)	0.912
GDM	8 (7.9%)	39 (9.8%)	0.573
Placenta previa	4 (4.0%)	13 (3.3%)	0.758
Fetal malformation	2 (2.0%)	6 (1.5%)	0.665
Macrosomia≥4000 g	3 (3.0%)	42 (10.5%)	0.018*
Low birthweight<2500 g	4 (4.0%)	18 (4.5%)	1.000

Note: 501 FET cycles with singleton live birth were included. In Group 1, three patients with twin live births were excluded. In Group 2, two patients with twin live births and one patient without perinatal outcome were excluded. Continuous data: mean ± SD. Categorical data: n (rate). Mann-Whitney tests were used in the continuous data and chi square tests or Fisher exact tests were used in the categorical data. *, *P*<0.05

*IVF*, in vitro fertilization, *ICSI* intracytoplasmic sperm injection, *GDM* gestational diabetes mellitus

*PCOS* polycystic ovary syndrome, *COH* controlled ovarian hyperstimulation

**Table 5:** The association between the two groups in perinatal outcomes among patients with singleton live birth

	OR (95%CI)	AOR (95%CI)	P(a)
Caesarean delivery	0.787 (0.384–1.614)	0.804 (0.379–1.707)	0.570
Male	0.859 (0.548–1.346)	0.897 (0.556–1.449)	0.658
Preterm birth<37 weeks	1.896 (0.651–5.521)	1.929 (0.620–6.003)	0.256
Gestational hypertension	1.050 (0.446–2.470)	0.894 (0.362–2.208)	0.807
GDM	1.256 (0.568–2.779)	1.166 (0.507–2.684)	0.717
Placenta previa	0.815 (0.260–2.554)	0.747 (0.226–2.473)	0.633
Fetal malformation	0.754 (0.150–3.792)	0.725 (0.136–3.867)	0.706
Macrosomia≥4000 g	3.832 (1.163–12.628)	3.886 (1.153–13.103)	0.029*
Low birthweight<2500 g	1.143 (0.378–3.454)	1.143 (0.349–3.735)	0.826

Note: Each perinatal outcome between the two groups was adjusted for age, *BMI* endometrial thickness, *ICSI* male infertility, endometriosis, *PCOS* protocol of COH, > 20 oocytes retrieved and progesterone > 1.5 ng/ml based on a multiple logistic regression model. *OR* odd ratio, *AOR* adjusted odd ratio

*GDM* gestational diabetes mellitus *, *P*<0.05

## Discussion

In this study, we analyzed 1025 FET cycles for single blastocyst transfer following freeze-all cycles and revealed that immediate FET cycles for blastocyst transfer following freeze-all cycles may result in pregnancy outcomes comparable to delayed FET cycles. However, postponement of FET cycles may increase the risk of macrosomia.

To our knowledge, a successful pregnancy depends on a complex process involving interactions between the endometrium and embryos [20]. With the popularity of freeze-all strategy, embryos are transferred into a more physiological intrauterine environment, which avoids asynchrony between endometrium receptivity and embryos development caused by supraphysiological hormonal levels during COH [7, 21]. The supraphysiological endocrine uterine environment and suboptimal endometrial development may lead to abnormal obstetric outcomes [8, 10, 11]. Meanwhile, from previous studies, fresh cycle may be associated with adverse perinatal outcomes compared with FET cycles, such as: perinatal mortality, low birthweight, preterm birth and so on [22, 23]. A meta-analysis included 13 cohort studies with 126,911 women also found singleton pregnancy after FET may have a better perinatal outcome compared with that after fresh cycles [24]. On the other hand, this strategy could significantly decrease the risk of OHSS. Jarvela et al. [10] also compared the serum progesterone and E_2_ levels in three groups (spontaneous pregnancies, fresh embryo transfer and FET) and found the serum progesterone and E_2_ levels were significantly higher in patients with fresh embryo transfer which negatively correlated with birthweight of the newborn. However, other studies supported that FET cycles could lead to cryo-injury which may influence the genetic potential of embryos and blastomeres. Degenerated blastomeres may influence the embryos implantation [25]. Therefore, the duration of supraphysiological hormonal levels and embryo freezing time on subsequent outcomes deserved our attention and enhance physician treatment confidence who hesitated whether start FET cycle immediately.

Postponement of FET cycles may be not only related to increase patients stress who eager to conceive as soon as possible, but also treatment burden. Maas et al. [26] found immediate FET cycles could result in higher pregnancy rates compared with delayed FET cycles. However, Ernest et al. [27] found high serum E_2_ concentrations in fresh IVF cycles may adversely affect implantation and pregnancy rates, but it did not affect subsequently implantation and pregnancy rates in FET cycles. Santos et al. [1] conducted a retrospective cohort study including 1183 first FET cycles. They found FET cycles performed immediately after a failed fresh embryo transfer had similar clinical pregnancy rate to those postponed to a later time which supported deferring FET may not improve pregnancy outcomes. Soon afterwards, they focused on patients with freeze-all strategy and also found immediately FET cycles after freeze-all strategy appeared to result in similar clinical pregnancy rates comparable to FET cycles deferred to a later time [28]. With the popularity of single blastocyst transfer, it could significantly decrease the risk of multiple pregnancies. From our study, we paid attention to the freeze-all cycles and subsequent single blastocyst transfer in artificially cycles which could reduce the effect of multiple embryo transfer and embryo transfer type on eventually outcomes. We found immediately FET may not increase the risk of abnormal pregnancy outcomes compared with delayed FET cycles which supported Santos’ viewpoint. However, their study did not focus on the potential carryover effect on perinatal outcomes, such as preterm birth, birth weight and so on. Therefore, we performed this study to further explore the effect of interval time after freeze-all cycles on perinatal outcomes and ensure the security of shortening FET intervals time.

Both the unadjusted and adjusted analyses showed that delayed FET may increase the risk of macrosomia without increased any other risks in perinatal outcomes when compared with immediate FET cycles. Some studies supported that abnormally high E_2_ levels could inhibit the normal trophoblastic invasion of the decidual and myometrial spiral arteries which may cause abnormal placentation and subsequent abnormal pregnancy outcomes [10, 13, 14, 29, 30]. What’ more, supraphysiological hormone may directly affect the peri-implantation embryo and implantation process by modulating the differentiation and invasive activity of the trophoblast cells [8]. From our study, it suggested that supraphysiological hormone levels during COH did not affect the outcomes of subsequent FET cycles after a menstrual cycle, but extended freezing time may increase the risk of macrosomia. A cumulative meta-analysis suggested that the increased risk of large for gestational age (LGA) and high birth weight was associated with frozen embryos [31]. Other studies also supported that FET singletons were at an increased risk of being born LGA and of being macrosomia [32, 33]. As we all know, frozen-thawed procedures could lead to cryo-injury. Capodanno et al. [25] supported that cryo-injury could influence the genetic potential of embryos and blastomeres. Therefore, we suspected whether delayed FET cycles may increase embryo freezing and in vitro time which eventually increased the risk of macrosomia. However, the number of singletons with macrosomia was small, so further research will be needed to elucidate it.

Although our present study was derived from a large sample size in freeze-all cycles and accounted for potentially confounding factors between the two groups, there still had certain limitations included the following: First, it used a retrospective and single-center study that increased the likelihood of bias. A prospective randomized controlled study could decrease selection bias. Second, the abnormal perinatal outcomes rate was calculated only including patients without abnormal complications and young age (≤40 years old) before ART which may decrease the risk of abnormal perinatal outcomes. Third, we only focused on patients with freeze-all cycles and the results were unable to account for all patients with IVF. Finally, some potential confounders were missing, such as smoking, abnormal pregnancy history and gestational weight gain, and so on. However, our studies adopted a new approach to divided patients into two group according to the interval time between oocyte retrieved and the day of first frozen-thawed blastocyst transferred which could more truly reflect the time of embryo in vitro and offer a more accurate evidence for infertility patients seeking for their next FET cycles. What’ more, we only included data from patients with single blastocyst transfer. It could decrease the impact of confounding factors and provide a critical evidence to elucidate the impact of FET time intervals after freeze-all cycles on subsequent pregnancy and perinatal outcomes.

## Conclusions

Our study clearly verified that delayed FET cycles for blastocyst transfer following freeze-all cycles may not improve the pregnancy outcomes. On the contrary, postponement of FET cycles may increase the risk of macrosomia. Therefore, we suggested FET cycles for blastocyst transfer should be done immediately to avoid adverse effects of delayed time on perinatal outcomes.

## Data Availability

The datasets used and analyzed during the current study are available from the corresponding author on reasonable request.

## Abbreviations

IVF: In-vitro fertilization
ICSI: Intracytoplasmic sperm injection
FET: Frozen-thawed embryos transferred
GDM: Gestational diabetes mellitus
OHSS: Ovarian hyperstimulation syndrome
COH: Controlled ovarian hyperstimulation
PGD: Preimplantation genetic diagnosis
PGS: Preimplantation genetic screening
LGA: Large for gestational age
PCOS: Polycystic ovary syndrome

## References

[1] Santos-Ribeiro S, Siffain J, Polyzos NP, van de Vijver A, van Landuyt L, Stoop D (2016). To delay or not to delay a frozen embryo transfer after a failed fresh embryo transfer attempt?. Fertil Steril.

[2] Sharma V, Allgar V, Rajkhowa M (2002). Factors influencing the cumulative conception rate and discontinuation of in vitro fertilization treatment for infertility. Fertil Steril.

[3] Chen YH, Wang Q, Zhang YN, Han X, Li DH, Zhang CL (2017). Cumulative live birth and surplus embryo incidence after frozen-thaw cycles in PCOS: how many oocytes do we need?. J Assist Reprod Genet.

[4] Davenport MJ, Vollenhoven B, Talmor AJ (2017). Gonadotropin-releasing hormone-agonist triggering and a freeze-all approach: the final step in eliminating ovarian Hyperstimulation syndrome?. Obstet Gynecol Surv.

[5] Stormlund S, Lossl K, Zedeler A, Bogstad J, Praetorius L, Nielsen HS (2017). Comparison of a 'freeze-all' strategy including GnRH agonist trigger versus a 'fresh transfer' strategy including hCG trigger in assisted reproductive technology (ART): a study protocol for a randomised controlled trial. BMJ Open.

[6] Zech J, Brandao A, Zech M, Lugger K, Neururer S, Ulmer H (2018). Elective frozen-thawed embryo transfer (FET) in women at risk for ovarian hyperstimulation syndrome. Reprod Biol.

[7] Weinerman R, Mainigi M (2014). Why we should transfer frozen instead of fresh embryos: the translational rationale. Fertil Steril.

[8] Royster GT, Krishnamoorthy K, Csokmay JM, Yauger BJ, Chason RJ, DeCherney AH (2016). Are intracytoplasmic sperm injection and high serum estradiol compounding risk factors for adverse obstetric outcomes in assisted reproductive technology?. Fertil Steril.

[9] Farhi J, Ben-Haroush A, Andrawus N, Pinkas H, Sapir O, Fisch B (2010). High serum oestradiol concentrations in IVF cycles increase the risk of pregnancy complications related to abnormal placentation. Reprod BioMed Online.

[10] Jarvela IY, Pelkonen S, Uimari O, Makikallio K, Puukka K, Ruokonen A (2014). Controlled ovarian hyperstimulation leads to high progesterone and estradiol levels during early pregnancy. Hum Reprod.

[11] Kalra SK, Ratcliffe SJ, Coutifaris C, Molinaro T, Barnhart KT (2011). Ovarian stimulation and low birth weight in newborns conceived through in vitro fertilization. Obstet Gynecol.

[12] Roque M, Lattes K, Serra S, Sola I, Geber S, Carreras R (2013). Fresh embryo transfer versus frozen embryo transfer in in vitro fertilization cycles: a systematic review and meta-analysis. Fertil Stertil.

[13] Khong TY, De Wolf F, Robertson WB, Brosens I (1986). Inadequate maternal vascular response to placentation in pregnancies complicated by pre-eclampsia and by small-for-gestational age infants. Br J Obstet Gynaecol.

[14] Labarrere CA, Althabe OH (1987). Inadequate maternal vascular response to placentation in pregnancies complicated by preeclampsia and by small-for-gestational-age infants. Br J Obstet Gynaecol.

[15] Chen ZJ, Shi Y, Sun Y, Zhang B, Liang X, Cao Y (2016). Fresh versus frozen embryos for infertility in the polycystic ovary syndrome. New Engl J Med.

[16] Ishihara O (2014). Impact of frozen-thawed single-blastocyst transfer on maternal and neonatal outcome: an analysis of 277,042 single-embryo transfer cycles from 2008 to 2010 in Japan (vol 101, pg 128, 2014). Fertil Steril.

[17] Huang Bo, Ren Xinling, Wu Li, Zhu Lixia, Xu Bei, Li Yufeng, Ai Jihui, Jin Lei (2016). Elevated Progesterone Levels on the Day of Oocyte Maturation May Affect Top Quality Embryo IVF Cycles. PLOS ONE.

[18] Zhu L, Xi Q, Zhang H, Li Y, Ai J, Jin L (2013). Blastocyst culture and cryopreservation to optimize clinical outcomes of warming cycles. Reprod BioMed Online.

[19] Kuwayama M, Gabor V, Kato O (2005). Highly efficient vitrification method for clinical cryopreservation of human oocytes. Reprod BioMed Online.

[20] Craciunas L, Gallos I, Chu J, Bourne T, Quenby S, Brosens JJ (2019). Conventional and modern markers of endometrial receptivity: a systematic review and meta-analysis. Hum Reprod Update.

[21] Zhu Q, Zhu J, Wang Y, Wang B, Wang N, Yin M (2019). Live birth rate and neonatal outcome following cleavage-stage embryo transfer versus blastocyst transfer using the freeze-all strategy. Reprod BioMed Online.

[22] Evans J, Hannan NJ, Edgell TA, Vollenhoven BJ, Lutjen PJ, Osianlis T (2014). Fresh versus frozen embryo transfer: backing clinical decisions with scientific and clinical evidence. Hum Reprod Update.

[23] Maheshwari A, Pandey S, Shetty A, Hamilton M, Bhattacharya S (2012). Obstetric and perinatal outcomes in singleton pregnancies resulting from the transfer of frozen thawed versus fresh embryos generated through in vitro fertilization treatment: a systematic review and meta-analysis. Fertil Steril.

[24] Zhao J, Xu B, Zhang Q, Li YP (2016). Which one has a better obstetric and perinatal outcome in singleton pregnancy, IVF/ICSI or FET?: a systematic review and meta-analysis. Reprod Biol Endocrinol.

[25] Capodanno F, De Feo G, Gizzo S, Nicoli A, Palomba S, La Sala GB (2016). Embryo quality before and after slow freezing: viability, implantation and pregnancy rates in 627 single frozen-thawed embryo replacement cycles following failure of fresh transfer. Reprod Biol.

[26] Maas KH, Baker VL, Westphal LM (2008). Optimal timing of frozen embryo transfer after failed IVF attempt. Fertil Steril.

[27] Ng EH, Yeung WS (2000). High serum oestradiol concentrations in fresh IVF cycles do not impair implantation and pregnancy rates in subsequent frozen-thawed embryo transfer cycles. Hum Reprod..

[28] Santos-Ribeiro S, Polyzos NP, Vuong TNL, Siffain J, Mackens S, Van Landuyt L (2016). The effect of an immediate frozen embryo transfer following a freeze-all protocol: a retrospective analysis from two centres. Hum Reprod.

[29] Imudia AN, Awonuga AO, Kaimal AJ, Wright DL, Styer AK, Toth TL (2013). Elective cryopreservation of all embryos with subsequent cryothaw embryo transfer in patients at risk for ovarian hyperstimulation syndrome reduces the risk of adverse obstetric outcomes: a preliminary study. Fertil Steril.

[30] Imudia AN, Awonuga AO, Doyle JO, Kaimal AJ, Wright DL, Toth TL (2012). Peak serum estradiol level during controlled ovarian hyperstimulation is associated with increased risk of small for gestational age and preeclampsia in singleton pregnancies after in vitro fertilization. Fertil Steril.

[31] Maheshwari A, Pandey S, Amalraj RE, Shetty A, Hamilton M, Bhattacharya S (2018). Is frozen embryo transfer better for mothers and babies? Can cumulative meta-analysis provide a definitive answer?. Hum Reprod Update.

[32] Luke B, Brown MB, Wantman E, Stern JE, Toner JP, Coddington CR (2017). Increased risk of large-for-gestational age birthweight in singleton siblings conceived with in vitro fertilization in frozen versus fresh cycles. J Assist Reprod Genet.

[33] Wei D, Liu JY, Sun Y, Shi Y, Zhang B, Liu JQ (2019). Frozen versus fresh single blastocyst transfer in ovulatory women: a multicentre, randomised controlled trial. Lancet..

